# NOX2 in autoimmunity, tumor growth and metastasis†

**DOI:** 10.1002/path.5175

**Published:** 2018-11-29

**Authors:** Anna Martner, Ebru Aydin, Kristoffer Hellstrand

**Affiliations:** ^1^ Department of Biomedicine, TIMM Laboratory Sahlgrenska Cancer Center, University of Gothenburg Gothenburg Sweden

**Keywords:** NOX2, metastasis, autoimmunity, immune checkpoint, chronic granulomatous disease, leukemia

## Abstract

Myeloid cell NADPH oxidase isoform 2 (NOX2) generates reactive oxygen species (ROS) that participate in defense against microbial pathogens. Humans with compromised NOX2‐mediated ROS formation develop chronic granulomatous disease characterized by recurrent bacterial and fungal infections. Additionally, impaired NOX2 function entails hyperactive lymphocytes and autoimmunity in humans and in murine models. The impact of NOX2 and ROS on cancer development is only partly explored. Recent research published in the *Journal of Pathology* showed that genetic depletion of any of the NOX2 subunits *Cyba*, *Cybb*, *Ncf1*, *Ncf2* and *Ncf4* reduced the formation of lung metastases following intravenous injection of murine tumor cells. These findings, together with the role of NOX2 in maintaining self‐tolerance, imply that NOX2 is a targetable immune checkpoint in cancer. In particular, the possibility of modulating NOX2 to improve lymphocyte‐mediated control of metastatic cells merits further investigation. © 2018 The Authors. *The Journal of Pathology* published by John Wiley & Sons Ltd on behalf of Pathological Society of Great Britain and Ireland.

## Introduction

NADPH oxidase isoform 2 (NOX2) is a multicomponent enzyme complex expressed almost solely in myeloid cells such as monocytes, macrophages and neutrophilic granulocytes. In the context of cancer, NOX2^+^ myeloid cells may infiltrate primary and metastatic tumors. Populations of leukemic myeloid cells also express functional NOX2. Although extensive literature is available on the impact of myeloid cells in cancer 1, the specific role of NOX2 and the consequences of its therapeutic targeting have only partially been explored. In a recent contribution to *The Journal of Pathology*, van der Weyden *et al* 2 reported that mice deficient in any of the five NOX2 subunits *Cyba*, *Cybb*, *Ncf1*, *Ncf2* or *Ncf4* were markedly less prone to develop lung metastases following intravenous injection of several histiotypes of tumor cells. These results confirm and extend previous results showing decreased pulmonary metastasis in *Cybb*‐deficient mice 3. Here we review aspects of NOX2 function in autoimmunity and malignancy with focus on experimental models of *Nox2* knockdown.

## NOX2

The only known action of NOX2 is to generate reactive oxygen species (ROS), which are short‐lived, toxic derivatives of oxygen that arise from the transfer of electrons over biological membranes. There is nomenclature confusion, in that NOX2 may refer to the membrane‐bound catalytic subunit of the NADPH oxidase (also known as CYBB or gp91^phox^), but may also refer to the entire oxidase. Here, we define NOX2 as the functional myeloid cell oxidase that is formed by the assembly of CYBB/gp91^phox^ with the membrane‐anchored CYBA/p22^phox^ and the cytosolic subunits NCF4/p40^phox^, NCF1/p47^phox^ and NCF2/p67^phox^ on the plasma membrane to generate extracellular ROS, or on a phagosome membrane to generate intracellular ROS.

Myeloid cells produce NOX2‐derived ROS as part of the innate immune defense against bacteria and other microorganisms. Additionally, ROS (including NOX2‐derived ROS) serve as signaling molecules by oxidizing thiol groups on proteins, thus modifying their function or activation status. Several antioxidant systems are operable within cells to detoxify ROS, but an imbalance between production and detoxification, referred to as ‘oxidative stress’, may be injurious to cellular components. Excessive ROS are thus implicated in several pathologies 4. Additionally, NOX2‐derived ROS released from myeloid cells may trigger dysfunction of neighboring cells, including antineoplastic lymphocytes such as natural killer (NK) cells and cytotoxic T cells 3, 5.

## NOX2 in pathogen defense and autoimmunity

The pivotal role of NOX2 in antimicrobial defense is highlighted by the striking susceptibility to bacterial and fungal infections observed in patients with chronic granulomatous disease (CGD), which is a rare genetic disorder caused by deficiency in one of the principal components of NOX2. The most common form of CGD is an X‐linked deficiency of *CYBB* 6. In addition to an insufficiency of pathogen clearance, patients with CGD frequently present with granulomas composed of activated macrophages and are at risk for developing autoimmunity 7. Recent genome‐wide association screens imply that variants of *NCF1* in humans, including copy number variations and single nucleotide polymorphisms, are strongly associated with major autoimmune diseases 8. These findings are in agreement with animal models, where genetic knockdown of *Ncf1* entailed autoimmunity with, for example, elevated autoantibody titers and kidney inflammation together with increased susceptibility to induction of autoimmune arthritis 9.

In coherence with the clinical features of CGD, van der Weyden *et al* 2 showed that mice with defective NOX2 function developed granulomas and an increased activation status of lymphocytes. In addition, *Cyba*‐deficient mice experienced a reduced lifespan due to breathing difficulties following lung granuloma formation and an increased incidence of lymphoma 2, presumably due to uncontrolled expansion of lymphocytes in the absence of NOX2‐mediated immunosuppression.

## Genetic knock‐down of NOX2 in cancer

ROS affect the initiation, growth and spread of cancer by multiple mechanisms and may therefore promote or suppress cancer development. Thus, ROS have been ascribed tumor‐promoting properties, for example by inducing DNA damage with ensuing mutations and by promoting proliferation and angiogenesis. By contrast, ROS have been suggested to exert tumor‐suppressive effects, such as induction of tumor cell apoptosis, autophagy and necroptosis 4, 10.

As ROS derive from multiple sources, including NOX enzyme isoforms and mitochondria during energy generation, genetic models of NOX2 deficiency have proven useful in defining the specific role of NOX2 in cancer. Kelkka *et al* 11 demonstrated that *Ncf1*‐deficient mice developed smaller melanoma tumors and fewer Lewis lung carcinoma tumors after implantation, which was accompanied by induction of pro‐inflammatory cytokines, although a distinct mechanism of enhanced immunity was not defined. However, *Ncf1* deficiency did not alter the growth of spontaneously arising prostate carcinoma (TRAMP) 11 or methylcholanthrene‐induced sarcomas 12. In the context of leukemia, human monocytic acute myeloid leukemia (AML) cells were reported to generate superoxide via NOX2 that promoted the survival of leukemic cells in xenografted mice. The proposed mechanism involved ROS‐induced transfer of mitochondria from stromal cells to AML blasts through AML‐derived tunneling nanotubes; these events did not occur in leukemic cells that were genetically deprived of NOX2 13. In addition, pharmacological inhibition of NOX2 using histamine dihydrochloride, which is used in conjunction with low‐dose interleukin‐2 for relapse prevention in the postchemotherapy phase of AML 14, reduced the expansion of xenografted human AML cells *in vivo* in a NOX2‐dependent manner 15.

**Figure 1:**
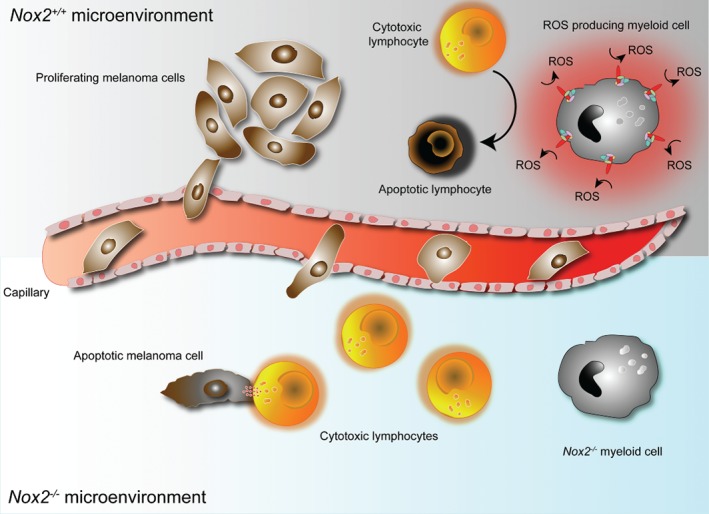
NOX2 in regulation of metastasis. Lung‐infiltrating myeloid cells express NOX2 that generate ROS, which, in turn, reduce the antimetastatic activity of adjacent lung‐infiltrating lymphocytes. In the absence of NOX2‐derived ROS, the antimetastatic function of lymphocytes is preserved.

The study by van der Weyden *et al* 2 supports that NOX2 significantly influences the process of metastasis as mice genetically deprived of any of the major NOX2 subunits, all of which are required for NOX2 functionality, consistently showed reduced lung metastasis after intravenous injection of tumor cells. These results confirm and extend reports showing reduced incidence of spontaneous metastasis after the removal of primary tumors in *Cybb* ^–/–^ mice 16 and reduced hematogenous melanoma metastasis in *Cybb* ^–/–^ mice 3. A similar reduction in metastasis was reported in mice treated with a NOX2 inhibitor. The latter study demonstrated that NOX2 inhibition facilitated NK cell‐mediated clearance of tumor cells, thus supporting that NOX2 exerts immunosuppression to promote metastasis 3. Additionally, Spiegel *et al* 17 recently reported that myeloid cells promote tumor metastasis, although the potential contribution of NOX2 was not investigated. Figure 1 provides a hypothetical model of the impact of NOX2 on metastasis based on these previous studies.

## Concluding remarks

Previous studies have shown that NOX2 is crucial for self‐tolerance, thus implying that NOX2 is an immune checkpoint. The studies presented here suggest that knock‐down of *NOX2* reduces metastasis via mechanisms that may involve amelioration of immune‐mediated clearance of metastatic tumor cells 2, 3, 16. However, the complexity regarding the role of ROS in cancer is significant and divergent results suggesting that ROS reduce metastasis in immune‐deficient mice have been reported 18. Collectively, these findings probably reflect that ROS may exert divergent effects depending on, for example, the source of ROS, the susceptibility of tumor cells to ROS toxicity, the phase of cancer progression and the sensitivity of immune effector cells to ROS‐induced immunosuppression. The possibility of specifically targeting NOX2 to reduce metastasis merits further investigation.

## Author contributions statement

All authors contributed to the preparation of this commentary.
